# Human papillomavirus in anal squamous cell carcinoma: an angel rather than a devil?

**DOI:** 10.3332/ecancer.2015.529

**Published:** 2015-04-29

**Authors:** Paola Simona Ravenda, Maria Giulia Zampino, Nicola Fazio, Massimo Barberis, Luca Bottiglieri, Susanna Chiocca

**Affiliations:** 1 Unit of Gastrointestinal and Neuroendocrine Tumours, European Institute of Oncology, Via Ripamonti 435, 20141 Milan, Italy; 2 Division of Histopathology and Molecular Diagnostics, European Institute of Oncology, Via Ripamonti 435, 20141 Milan, Italy; 3 Department of Experimental Oncology, European Institute of Oncology, Via Ripamonti 435, 20141 Milan, Italy

**Keywords:** anal squamous cell carcinoma, anal cancer, human papillomavirus, HPV-16

## Abstract

Anal cancer is a rare disease with an increasing incidence worldwide but, unfortunately, even today the scientific community still has a limited knowledge and limited options of treatment.

More than 50% of patients with anal cancer presenting at diagnosis with locoregional disease have good chances of cure with chemoradiotherapy (CT–RT). However, once patients develop metastatic spread, the prognosis is very poor.

Human papillomavirus (HPV) is present in more than 80% of anal cancers and while multiple etiologic connections between HPV infection and anal cancer have already been well elucidated, its prognostic and/or predictive role is currently under investigation, especially among immunocompetent patients affected by this disease.

In a single-institutional set, we have retrospectively analysed clinical data of 50 consecutive cases homogeneously treated with CT–RT for stage I–III anal squamous cell carcinoma. We found that HPV-positive anal cancers had a statistically significant improved five-year disease-free survival (DFS) compared to HPV-negative group. These findings could be explained by an increased chemo/radiosensitivity of HPV-positive tumours. Further efforts should be directed towards a better understanding of HPV-related oncogenesis and towards designing novel tailored strategies for the management of this disease both in terms of prevention and treatment.

## Background and discussion

Anal carcinoma (AC) is a rare disease in the general population, being estimated at about 1.5 cases per 100,000 people per year worldwide, but with an increasing incidence particularly in women [1, 2].

Several biomolecular and epidemiologic studies have clearly elucidated a strong etiologic connection between AC and high-risk genotypes of HPV [3, 4], and indeed HPV infection is detected in between 86% and 97% of all AC cases [5].

Factors increasing the risk of HPV infection and/or modulating host response and the persistence of this infection appear to affect the epidemiology of this tumour. Anal intercourse and a high lifetime number of sexual partners increase the risk of persistent HPV infection in men and women, leading eventually to malignancy. Other important risk factors include human immunodeficiency virus (HIV), immunosuppression in transplant recipients, use of immnunosuppressants, a history of other HPV-related cancers, autoimmune disorders, social deprivation, and cigarette smoking [6].

Not surprisingly, AC is no longer a rare disease within specific epidemiologic categories, since among HIV seropositive men the incidence of AC increases to 75–135 per 100,000 and is also higher among HIV seropositive women [6]. Therefore, in this regard, AC is one of the most representative examples of human cancers that emphasises how much the immune system and the process of tumourigenesis are inextricably linked.

On the other hand, few studies have so far evaluated the prognostic or predictive role of HPV in AC, especially among the general population and immunocompetent patients.

Based on these considerations we have retrospectively reviewed the data of a series of 50 consecutive and immunocompetent cases, treated in our institution between 2000 and 2012. These patients had AJCC (American Joint Committee on Cancer) stage I–III histologically-proven anal squamous cell carcinoma, and had undergone standard treatment with concurrent chemo-radiotherapy (CT–RT). In all patients the presence of HPV infection was assessed by a polymerase chain reaction (PCR) analysis on the tumour biopsy provided at the time of diagnosis before treatment. Genomic DNA was extracted from three 10-μm-thick paraffin sections using QIAamp DNA FFPE Tissue kit (Qiagen, Hiden, Germany), following the manufacturer’s instructions, then 200 ng of DNA were amplified using L1 primers and GP5+/GP6+ primers. For all the PCR procedures, a positive and a negative control were provided. All the positive cases were confirmed in a second round of amplification. The PCR products were detected by electrophoresis with a 2% agarose gel and stained with ethidium bromide and in the presence of HPV infection, we proceeded to do the genotyping by testing the presence of high-risk HPV16, 18, 31, 33, and 45, using specific primers. Inclusion and exclusion criteria as well as the details on concurrent CT–RT are extensively reported elsewhere [7].

In our study we found that 84% of patients were HPV-positive and, among these HPV genotype 16 was detected in 90% of cases. HPV-positive patients had more advanced disease at diagnosis compared to HPV-negative patients in terms of nodal involvement affecting TNM clinical stage.

In particular, within HPV-positive group 2.4% of patients had stage I, 23.8% stage II, 45.2% stage IIIA, 28.6% stage IIIB, and within HPV-negative group 0% of patients had stage I, 62.5% stage II, 37.5% stage IIIA, 0% stage IIIB.

In this regard, in the statistical analysis, we decided to apply an adjustment for clinical stage in multivariate analysis since the HPV-positive patients presented a higher rate of nodal involvement [7].

After a median follow-up of four years, we found that five-year DFS in HPV-positive and HPV-negative patients was 92.5% and 50% respectively (p < 0.01). Five-year overall survival (OS) in HPV-positive and HPV-negative patients was 93.3% and 66.7%, respectively (p = 0.12). In conclusion, patients with HPV-positive AC had a strong statistically significant improved DFS compared to that in HPV-negative patients; the same trend was observed for OS, but statistical significance was not reached.

Despite its small sample size and retrospective nature, this study shows that HPV infection in AC is a positive prognostic factor for DFS. This could be explained by the better response of HPV-positive AC to CT–RT, as already shown in HPV-positive oropharyngeal squamous cell carcinoma [8, 9].

Certainly a greater patient sample size and longer follow-up might also show in the near future a significant difference in OS between HPV-positive and negative AC.

From our perspective, the most intriguing and challenging question is whether HPV-positive AC and HPV-negative AC are two completely different malignancies sustained by different biomolecular features and genetic alterations. Further research should be directed towards investigating which specific pathways are selectively altered in anal cancers which are HPV-positive and those which are HPV-negative, by adopting a comparative approach. This should lead to a better understanding of HPV-related oncogenesis and to the design of novel and personalised tailored strategies for the management of this disease both in terms of prevention and treatment.

Preclinical models as faithful as possible to the true HPV-related tumour cell biology should be the right place from which experimental studies can embark upon their challenging road.

Currently the vast majority of modern studies, including analysis of E6 and E7 mRNA [10, 11], PIK3CA mutations [12, 13], and inhibition of aberrant cap-dependent protein translation [14, 15] are essentially applied to head and neck and cervical cancer models. Instead we believe that, because of shared underlying HPV biology, there is an opportunity for collaborative development of research strategies among investigators across the spectrum of HPV-related malignancy. Indeed in our centre we are building upon a new multidisciplinary approach in order to connect and guide scientific and clinical efforts within a common network for prevention and treatment of these diseases.

## Conclusion

Despite its small sample size and retrospective nature, this study shows that HPV infection in anal cancer is a positive prognostic factor for disease-free survival. This could be explained by the better response of HPV-positive anal cancer to chemo-radiotherapy, as already shown in HPV-positive oropharyngeal squamous cell carcinoma.

From our perspective, the most intriguing and challenging question is whether HPV-positive anal cancer and HPV-negative anal cancer are two completely different malignancies sustained by different biomolecular features and genetic alterations. Further research should be directed towards investigating which specific pathways are selectively altered in anal cancers which are HPV-positive and those which are HPV-negative, by adopting a comparative approach. This should lead to a better understanding of HPV-related oncogenesis and to the design of novel and personalised tailored strategies for the management of this disease both in terms of prevention and treatment.

## Conflicts of interest

The authors declare that they have no conflicts of interest.
